# The effect of naloxone treatment on opioid-induced side effects: A meta-analysis of randomized and controlled trails

**DOI:** 10.1097/MD.0000000000004729

**Published:** 2016-09-16

**Authors:** Feifang He, Yilei Jiang, Li Li

**Affiliations:** a Department of Anesthesiology & Pain Management, Sir Run Run Shaw Hospital, Zhejiang University School of Medicine, Hangzhou, Zhejiang, China; b Department of Anesthesiology, The Children's Hospital, Zhejiang University School of Medicine, Hangzhou, Zhejiang, China; c Department of General Practice, Sir Run Run Shaw Hospital, Zhejiang University School of Medicine, Hangzhou, Zhejiang, China.

**Keywords:** meta-analysis, naloxone, nausea, opioid-induced side effects, pruritus, vomiting

## Abstract

**Background:**

To evaluate the effects of naloxone on opioid-induced side effects, the present meta-analysis was constructed.

**Methods:**

Electronic databases including PubMed, EMBASE, and CNKI (China National Knowledge Internet) were used for literature search. Studies on comparison of opioid-side effects between naloxone-treated group and placebo or normal saline-related group were included in the meta-analysis. Heterogeneity analysis was performed with Chi-square and *I*^2^ test. Pooled analysis was based on fixed-effects model, if heterogeneity between the eligible studies was negligible (*I*^2^ < 50%, *P* > 0.05), otherwise, random-effects model was used. Sensitivity analysis was applied to assess the robustness of the results and publication bias was evaluated by Begg and Egger test.

**Results:**

Thirteen studies including 1138 patients were included in the meta-analysis. Pooled analysis indicated that naloxone could significantly reduce the occurrence of pruritus (RR [risk ratio] = 0.252, 95% CI [confidence interval] = 0.137–0.464), nausea (RR = 0.323, 95% CI = 0.245–0.428), and vomiting (RR = 0.338, 95% CI = 0.192–0.593) which were induced by opioids. However, naloxone did not relieve pain (standardized mean difference [SMD] = −0.052, 95% CI = −0.453 to 0.348) and somnolence (RR = 0.561, 95% CI = 0.287 to 1.097) in patients received opioid treatment. Additionally, there were no significant publication bias between the included studies (Begg test, *P* = 0.602; Egger test, *P* = 0.388).

**Conclusion:**

Addition of naloxone might act as an effective treatment for prophylaxis of opioid-induced pruritus, nausea, and vomiting in clinical practice.

## 1 Introduction

Pain management is a common worldwide healthy problem.^[1]^ Opioid is an effective treatment for moderate-to severe cancer-related and noncancer pain.^[2,3]^ The utilization of opioid has sharply increased in recent years in most parts of the world.^[4]^ However, various side effects are reported to be significantly associated with opioid therapy, including pruritus, nausea, vomiting, constipation, urinary retention, respiratory depression, and sedation.^[5,6]^ Although not life-threatening, the side effects are unpleasant which may lead to patients’ discomfort, decreased quality of life.^[7]^ The mechanism of opioid-induced side effects has not been completely explained. Some studies demonstrated that mu-opioid receptor may contribute to the occurrence of side effects.^[5,8,9]^

Naloxone, an opioid antagonist, has long been used to diagnose and manage respiratory depression related to opioid overdose.^[10]^ Recently, some studies indicated that the combined application of naloxone and opioid may reduce opioid-related side effects. A study carried out by Xiao et al^[11]^ proved that naloxone infusion could prevent the acute opioid tolerance, provide a quicker recovery of bowel function, and reduce the length of hospital stay after open colorectal surgery. In a retrospective study, naloxone application was proved to be a measure to track opioid safety in children, identify contributing factors, and formulate preventive strategy to reduce the risk for opioid-induced respiratory depression.^[12]^ However, some studies hold different opinions. Cepeda et al had reported that adding low doses of naloxone to a morphine patient-controlled analgesia solution increased opioid requirement and pain. Moreover, the incidence of side effects had not reduced.^[13]^ In the study of Bijur et al,^[14]^ the similar conclusion was obtained. There is no agreement on this issue, so the current meta-analysis was conducted to evaluate the effects of naloxone on opioid-induced side effects.

In the present study, we aimed to evaluate the effects of naloxone on side effects induced by opioid via a meta-analysis. Studies for comparison of opioid-induced side effects between naloxone-treated group and placebo or normal saline (NS) therapeutic group were included in the present study. Meta-analysis was conducted to compare the occurrent of pruritus, nausea, vomiting, and somnolence in study groups. The present study may provide a reference for naloxone application in prevention of opioid-induced side effects in clinical practice.

## 2 Materials and methods

### 2.1 Search strategy

All authors reviewed the results and approved the final version of the manuscript. This study was approved by the Ethics Committee of the Sir Run Run Shaw Hospital.

In order to identify eligible studies for inclusion in the present meta-analysis, we did a broad search in the following databases, PubMed, EMBASE, and CNKI (China National Knowledge Internet). The search strategy included the key words: “naloxone” AND “opioid” AND “side effects” OR “constipation” OR “nausea” OR “pruritus” OR “vomiting.” In addition, reference lists of the included studies were checked for eligible researches. No language restriction was applied.

### 2.2 Eligibility criteria

The studies were considered to be included based on the following criteria: The study design was a cohort study, including test group and control group. Patients in both test group and control group received opioid therapy. The only difference between test group and control group was that patients in test group received naloxone treatment. All the included studies were based on adult population. The patients had not been treated with opioid drugs. The data of outcome measures for test group and control group were shown in the articles. For different reports of the same clinical trail, the recent study was included.

### 2.3 Data extraction

The extracted data included the first author, year, generally demographic characteristics of the included patients, study patients group, therapeutic regimen, group information, and outcome measures.

### 2.4 Statistical analysis

Cochrane assessment tool was used to evaluate the risk of bias in the eligible studies according to the previous description.^[15]^ Meta-analysis for dichotomous data was evaluated with risk ratio (RR) with 95% confidence interval (CI), while continuous variables were analyzed by standardized mean difference (SMD) with 95% CI. Statistical heterogeneity was assessed by Chi-square analysis and *I*^2^ test. Fixed-effects model was used when there was no obvious heterogeneity (*I*^2^ > 50%, *P* < 0.05). Otherwise, meta-analysis was based on random-effects model. The robustness of the analysis results was detected by sensitivity analysis. Begg test and Egger test were used for publication bias analysis. *P* < 0.05 was considered statistically significant. The current meta-analysis was performed in Stata 12.0 software (Stata Corp LP, College Station, Texas, USA).

## 3 Results

### 3.1 Selection process

**Figure 1:**
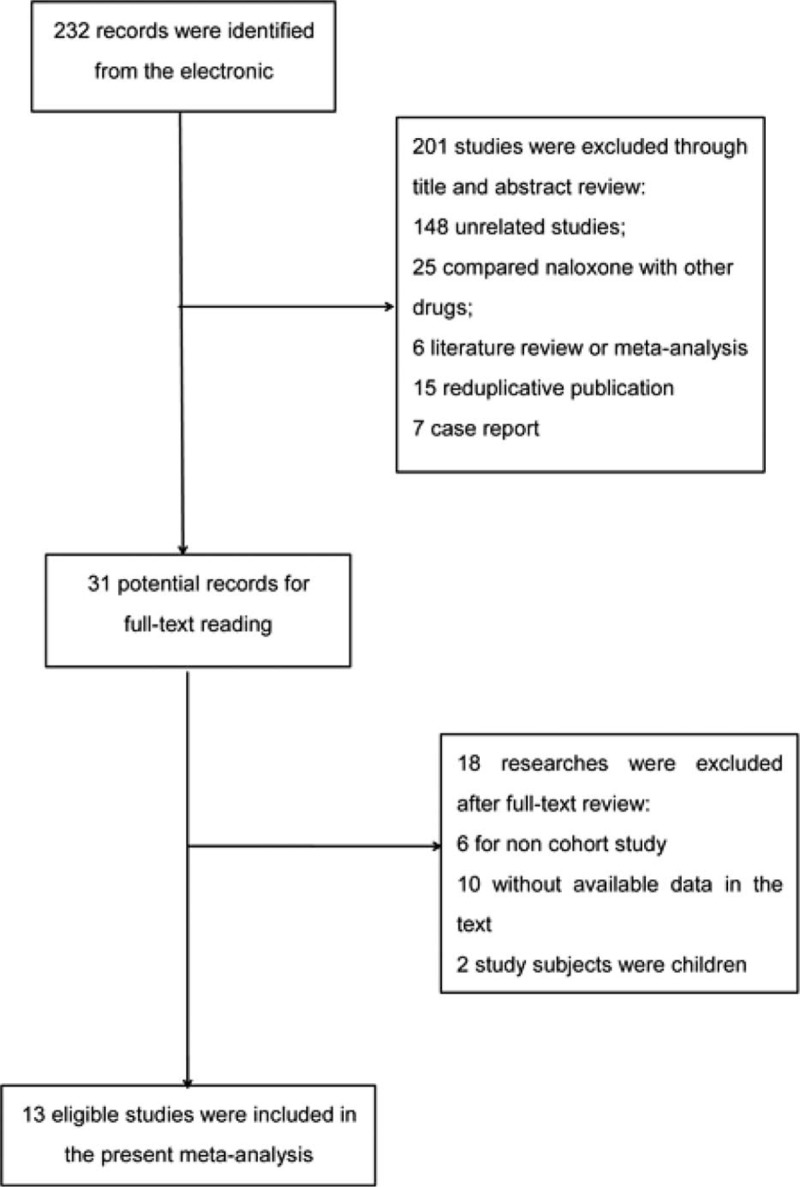
Selection process. A broad selection was done in PubMed, EMBASE, and CNKI and 13 eligible studies were included in the present meta-analysis.

Through a broad selection for the electronic databases, 232 studies were identified. After title and abstract review, 201 records were excluded: 148 unrelated studies, 6 for literature review or meta-analysis, 15 for repeated publication, 7 for case report, 25 for comparing naloxone with other drugs. Thirty-one potential records were needed to be identified via full-text reading. Then 18 researches were excluded: 6 for noncohort study, 10 for without available data, 2 for research on children. Finally, 13 studies meeting the inclusion criterion were included in the present meta-analysis.^[16–28]^ The selection process are shown in Fig. 1.

In the 13 eligible studies, 1138 patients were included. Six hundred fifteen patients received naloxone treatment were in test group, while 523 patients treated with NS or placebo therapy were in control group. The clinical characteristics of the patients were similar between test group and control group. Baseline characteristics of the included studies are shown in Table 1.

**Table 1:**
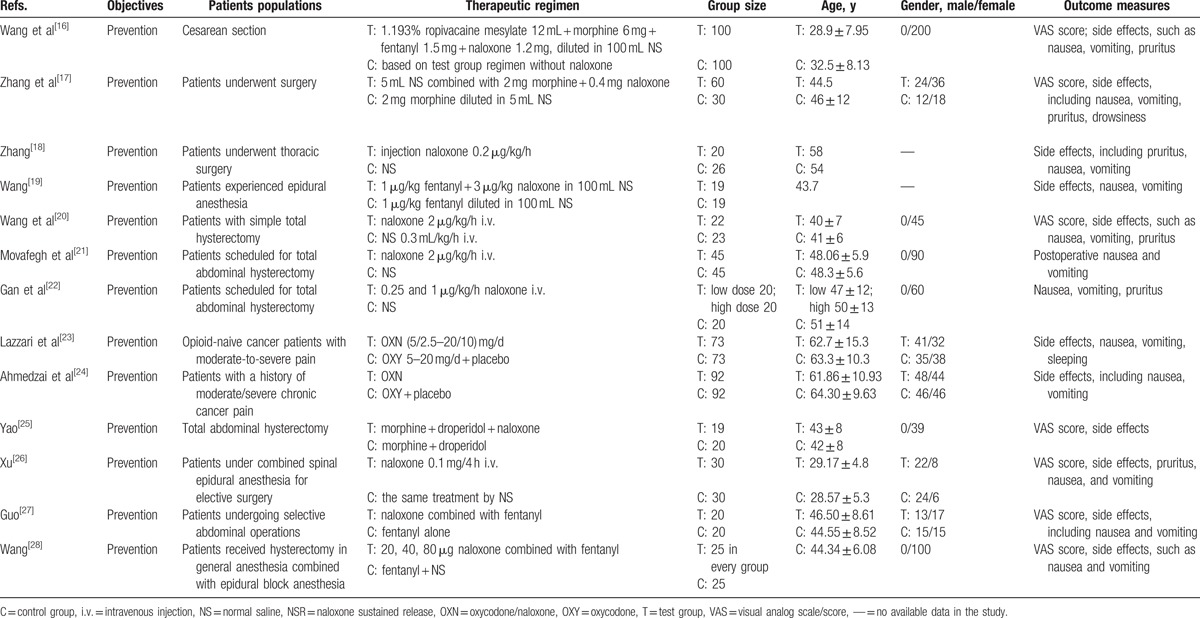
Baseline characteristics of the included studies.

### 3.2 Evaluation for quality of the included studies

**Figure 2:**
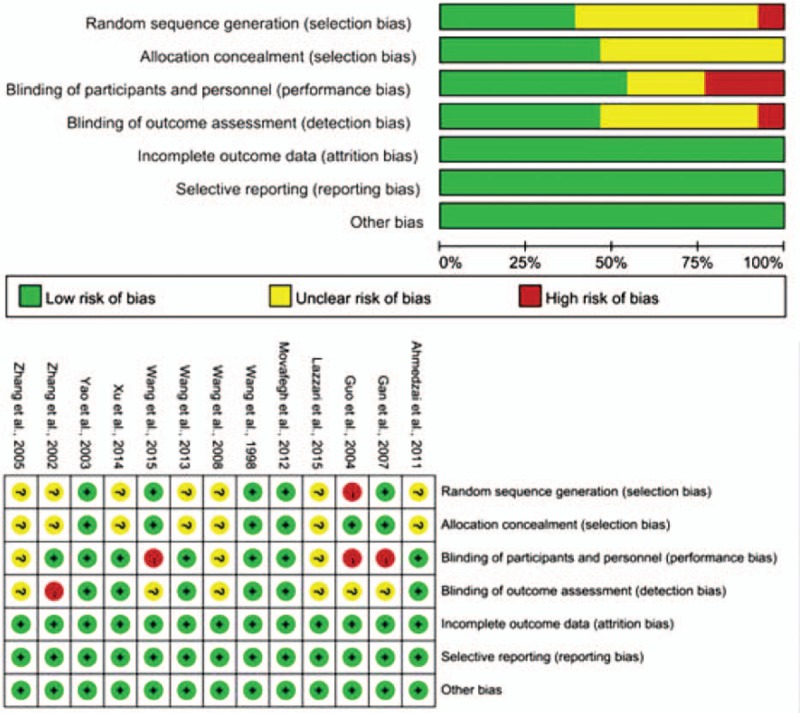
Risk of bias evaluation for the included studies. Analysis results indicated that the included records were with high quality.

The quality of the included studies was evaluated by Cochrane assessment tool. Cochrane assessment system included random sequence generation, allocation concealment, blinding of the participants, blinding of treatment providers, intention to treat, selective reporting, comparable study groups, and other bias. Evaluation results for included studies quality indicated that all of the eligible studies in the current meta-analysis were with high quality and the meta-analysis results were true and credible (Fig. 2).

### 3.3 Effects of naloxone for opioid-induced pruritus

**Figure 3:**
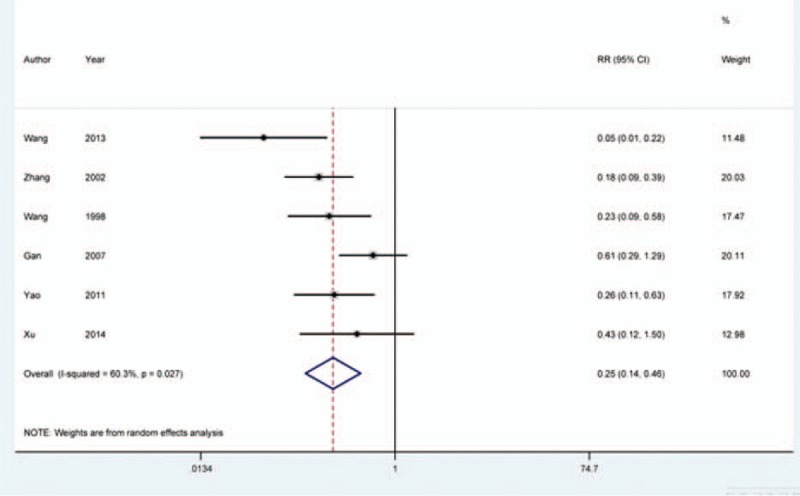
Meta-analysis for effects of naloxone on pruritus induced by opioid. Pooled analysis was based on random-effects model and the results indicated that occurrence rate of opioid-induced pruritus was significantly lower in naloxone-treated group than that in the control group (RR = 0.252, 95% CI = 0.137–0.464, *P* = 0.000).

Six eligible studies discussed the effects of naloxone on opioid-induced pruritus.^[16,17,20,22,25,26]^ Heterogeneity analysis indicated that there was significant heterogeneity between the included studies (*I*^2^ = 60.3%, *P* = 0.027). Therefore, meta-analysis was based on random-effects model. Analysis results indicated that the occurrence rate of opioid-induced pruritus was significantly lower in naloxone group than that in the control group (RR = 0.252, 95% CI = 0.137–0.464, *P* = 0.000) (Fig. 3).

### 3.4 Meta-analysis for nausea caused by opioid

**Figure 4:**
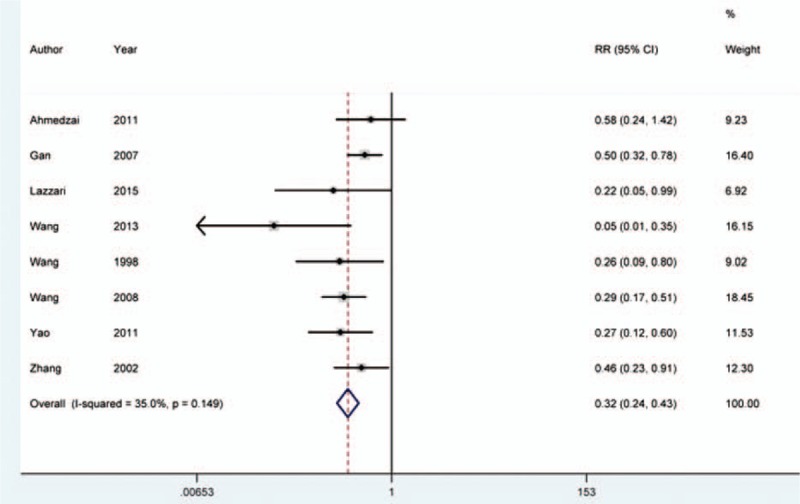
Analysis for nausea induced by opioid. Analysis based on fixed-effects model suggested that addition of naloxone could prevent incidence of nausea in patients received opioid treatment (RR = 0.323, 95% CI = 0.245–0.428, *P* = 0.000).

We conducted meta-analysis for opioid-induced nausea and eight studies were included.^[16,17,20,22–25,28]^ The meta-analysis was analyzed by fixed-effects model, for no obvious heterogeneity presented in the eligible studies (*I*^2^ = 35.0%, *P* = 0.149). Analysis results demonstrated that patients in control group were more likely to undergo nausea than those in the test group (RR = 0.323, 95% CI = 0.245–0.428, *P* = 0.000) (Fig. 4).

### 3.5 Pooled analysis for vomiting

**Figure 5:**
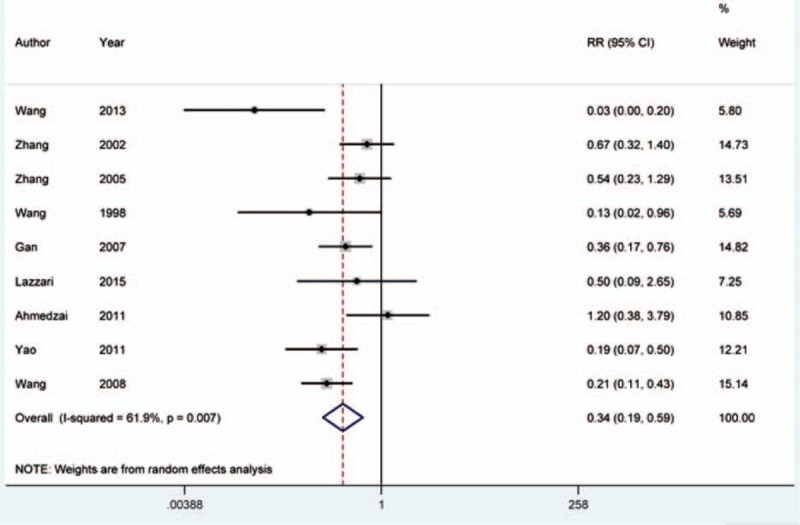
Meta-analysis conducted for opioid-induced vomiting. The incidence rate of vomiting caused by opioid was significantly lower in naloxone-treated group than that in the control group (RR = 0.338, 95% CI = 0.192–0.593, *P* = 0.000).

The dichotomous data for occurrence of vomiting was extracted from 9 eligible records.^[16–18,20,22–25,28]^ Chi-square and *I*^2^ test demonstrated that there was significant heterogeneity between the included studies (*I*^2^ = 61.9%, *P* = 0.007). Meta-analysis conducted with random-effects model indicated that naloxone treatment could lower the rate of vomiting (RR = 0.338, 95% CI = 0.192–0.593, *P* = 0.000) (Fig. 5).

### 3.6 Analysis for nausea and vomiting

Five of the included studies compared the occurrence rate of nausea and vomiting between test group and control group.^[19–21,26,27]^ Heterogeneity analysis indicated that the heterogeneity between the included studies was negligible (*I*^2^ = 44%, *P* = 0.128). The present meta-analysis based on fixed-effects model indicated that the incidence rate of nausea and vomiting was lower for patients treated with naloxone, compared with those in the control group (RR = 0.310, 95% CI = 0.173–0.556, *P* = 0.000).

### 3.7 Meta-analysis for somnolence

The data for effects of naloxone on opioid-induced somnolence was reported in 2 of the included studies.^[17,23]^ Fixed-effects model was applied for meta-analysis for no obvious heterogeneity presented (*I*^2^ = 0.0%, *P* = 0.607). Pooled analysis suggested that naloxone treatment did not influence the occurrence rate of somnolence (RR = 0.561, 95% CI = 0.287–1.097, *P* = 0.091).

### 3.8 Effects of naloxone on pain intensity

**Figure 6:**
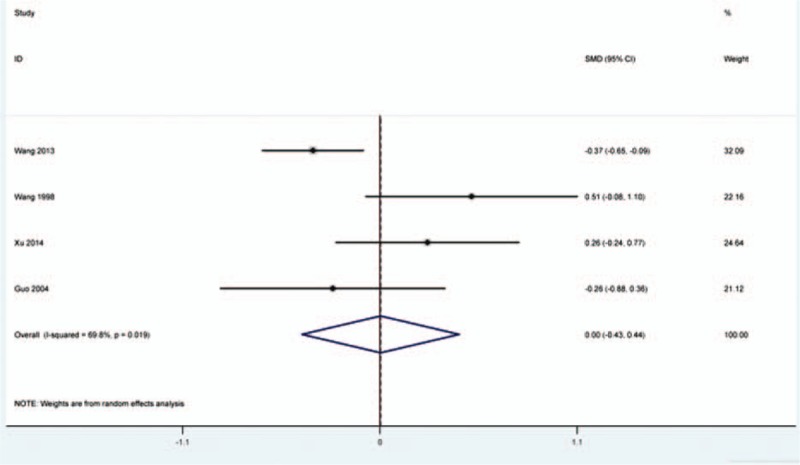
Effects of naloxone on pain intensity. Meta-analysis indicated that VAS score was similar between naloxone-treated group and placebo or NS group for patients received opioid treatment (SMD = −0.052, 95% CI = −0.453 to 0.348, *P* = 0.798).

VAS (visual analog scale/score) was applied to evaluate postoperative pain for patients included in the present study. The data of VAS score was reported in 4 records.^[16,19,26,27]^ Significant heterogeneity analysis indicated that obvious heterogeneity presented between the included studies (*I*^2^ = 69.8%, *P* = 0.019). Based on random-effects model, analysis indicated that VAS score was similar between test group and control group (SMD = 0.003, 95% CI = −0.434 to 0.441, *P* = 0.988) (Fig. 6).

### 3.9 Sensitivity analysis

One study was removed at each time and the results did not change obviously, indicating the robustness of analysis results.

### 3.10 Publication bias

**Figure 7:**
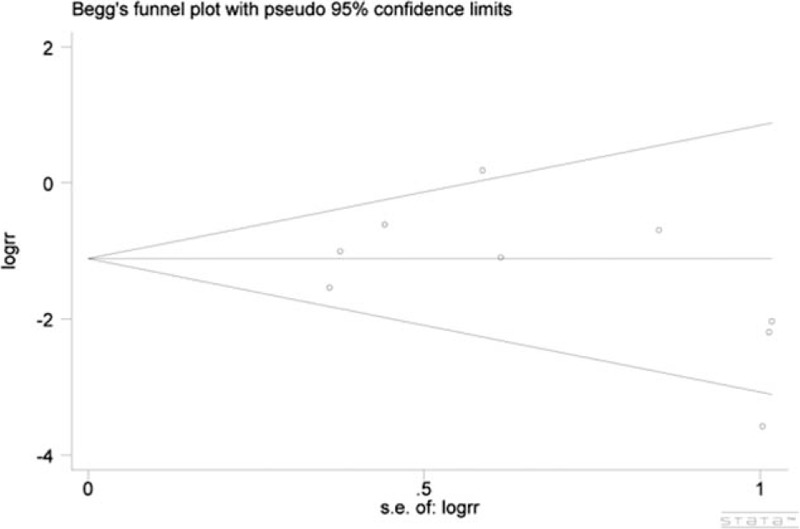
Begg funnel plot for meta-analysis of vomiting. The results indicated that there was no publication bias in the included studies (Begg test, *P* = 0.602; Egger test, *P* = 0.388).

In the present meta-analysis, Egger test and Begg test were applied for publication bias analysis. The results indicated that there were no significant publication bias between the included studies (Begg test, *P* = 0.602; Egger test, *P* = 0.388) (Fig. 7).

## 4 Discussion

Opioid is an effective treatment for pain due to surgery, labor, and disease. However, opioid induced several adverse effects, including pruritus, nausea, vomiting, and constipation. In the previous studies, some therapeutic measures were taken to manage opioid-induced side effects. Jamal et al^[29]^ had proved that lubiprostone could significantly improve opioid-induced constipation in patients with chronic noncancer patient, without adverse effects. A system review conducted by Jannuzzi indicated that nalbuphine was an effective treatment for opioid-induced pruritus in patients receiving opioids for acute pain related to surgery or childbirth. Nalbuphine may serve as a first-line treatment for opioid-induced pruritus.^[30]^ A questionnaire survey among Japanese physicians suggested that prophylactic antiemetics, most commonly prochlorperazine, were used for prophylaxis of opioid-induced nausea and vomiting in clinical practice.^[31]^ In the present meta-analysis, we demonstrated that naloxone could prevent the occurrence of pruritus, vomiting, and nausea induced by opioids, but it had no efficacy on pain intensity and somnolence.

Pruritus was a common side effect caused by opioid. Although it was not life-threatening, pruritus could reduce quality of life of patients.^[32]^ In the present study, 6 included studies discussed the effects of naloxone on opioid-induced pruritus. Pooled analysis indicated that addition of naloxone could prevent the incidence of opioid-induced pruritus. This conclusion was consistent with the previous studies. A quantitative systematic review of randomized trials indicted that naloxone was efficacious in the prevention of opioid-induced pruritus.^[8]^ However, the mechanism for naloxone treating for opioid-induced pruritus had not been completely understood. In addition, the effects of naloxone on treatment of opioid-induced pruritus were needed to be identified in clinical practice.

In the present study, we conducted meta-analysis for occurrence rate of nausea and vomiting induced by opioid in test group and control group. Analysis results indicated that naloxone was superior to placebo and NS in the prevention of vomiting and nausea caused by opioid. It was reported that among patients undergoing general anesthesia with opioids, most of them were likely to suffer from postoperative nausea and vomiting.^[33]^ Nausea and vomiting were distressing in patients, which could reduce satisfaction of patients, prolong hospital stay, and increase costs.^[34]^ Preventing the incidence of nausea and vomiting may significantly improve quality of life for patients. The efficacy of naloxone for preventing opioid-induced nausea and vomiting was identified in the present meta-analysis, which may be widely used in clinical practices.

In addition, we found that naloxone did not influence the occurrence of somnolence and pain intensity. Some of the previous records had reported that addition of naloxone could reduce opioids consumption. However, a study scheduled by Guerriero et al^[35]^ demonstrated that addition of low-dose naloxone could significantly relieve pain for chronic noncancer pain in older patients. The difference may be due to the small literature sample size in the present meta-analysis. In the next study, the effects of naloxone on VAS score were needed to be confirmed by large-sample randomized controlled trials.

There were still several limitations in the present study. Firstly, the patient populations included in the present meta-analysis were different. The patients in the selected studies included puerpera, patients undergoing surgery and cancer patients. The variations in patient populations may influence the overall results. Secondly, the management of naloxone was different between the included studies. Differences in administration route and dose of naloxone might lead to certain errors in the analysis results. In addition, various opioid available may also influence the pooled analysis. Finally, in the present study, we proved that naloxone could obviously prevent side effects induced by opioids, including pruritus, nausea, and vomiting. However, if naloxone could be applied for treatment of opioid-induced side effects were needed to be identified by a larger-sample of studies.

In conclusion, addition of naloxone is an effectively therapeutic strategy to prevent opioid-induced side effects, such as pruritus, nausea, and vomiting. However, naloxone cannot relieve pain intensity and somnolence for patients treated with opioids. Naloxone may be an effective measure for prophylaxis of opioid-induced pruritus, nausea, and vomiting in clinical practice.
